# Prevalence, patterns, and predictors of meditation use among US adults: A nationally representative survey

**DOI:** 10.1038/srep36760

**Published:** 2016-11-10

**Authors:** Holger Cramer, Helen Hall, Matthew Leach, Jane Frawley, Yan Zhang, Brenda Leung, Jon Adams, Romy Lauche

**Affiliations:** 1 Department of Internal and Integrative Medicine, Kliniken Essen-Mitte, Faculty of Medicine, University of Duisburg-Essen, Essen, Germany; 2 Australian Research Centre in Complementary and Integrative Medicine (ARCCIM), Faculty of Health, University of Technology Sydney, Sydney, New South Wales, Australia; 3 School of Nursing and Midwifery, Monash University, Frankston, VIC, Australia; 4 School of Nursing & Midwifery, University of South Australia, Adelaide, South Australia; 5 Department of Family and Community Medicine, Texas Tech University Health Sciences Center, Lubbock, Texas, USA; 6 University of Lethbridge, Lethbridge, Alberta, Canada

## Abstract

Emerging evidence suggests substantial health benefits from using meditation. While there are some indications that the popularity of meditation is increasing, little is known about the prevalence, patterns, and predictors of meditation use in the general population. In this secondary analysis of data from the 2012 US National Health Interview Survey (NHIS) (n = 34,525), lifetime and 12-month prevalence of meditation use were 5.2% and 4.1%, respectively. Compared to non-users, those who had used meditation in the past 12 months were more likely to be 40–64 years, female, non-Hispanic White, living in the West, at least college-educated, not in a relationship, diagnosed with one or more chronic conditions, smoking, consuming alcohol and physically active. Meditation was mainly used for general wellness (76.2%), improving energy (60.0%), and aiding memory or concentration (50.0%). Anxiety (29.2%), stress (21.6%), and depression (17.8%) were the top health problems for which people used meditation; 63.6% reported that meditation had helped a great deal with these conditions. Only 34.8% disclosed their use of meditation with a health provider. These findings indicate that about 9.3 million US adults have used meditation in the past 12 months; and that mental health problems were the most important reason for meditation use.

Meditation is a mind-body practice originating from eastern traditions, with a history spanning more than 3000 years. Although there are a variety of approaches, meditation fundamentally involves undertaking a set of intentional practices that lead to increased awareness, greater presence, and a more integrated sense of self^1^.

Emerging evidence indicates that meditation may generate substantial health benefits for users. For instance, studies have shown that meditation may be a useful adjunct to the treatment of mental health problems, particularly mood and anxiety disorders^2^,^3^,^4^. Evidence also suggests benefits of meditation for people suffering from physical conditions, including hypertension^5^, insomnia^6^, irritable bowel syndrome^7^ and symptoms related to epilepsy, premenstrual syndrome and menopause^2^. Importantly, meditation is considered to be a generally safe practice; while isolated reports suggest that meditation may aggravate some mental health problems^8^, the strength of this evidence is very low.

The positive safety profile of meditation, broad indications for use, evidence of effectiveness for many conditions, and ease of use, might infer a high prevalence of meditation use in the general population. Indeed, some authorities have suggested that the popularity of meditation is increasing^2^. However, few studies have specifically measured the prevalence of meditation use; in fact, most studies exploring the utilization of complementary and alternative medicine have either excluded meditation from their list of therapies, not presented data on meditation use, or grouped meditation under a broad category, such as relaxation therapies, which is unhelpful. The small number of studies reporting meditation use indicate a prevalence rate of between 10.2% (Barnes, *et al*.^9^) and 17.2%^10^; notwithstanding, these estimates are somewhat dated (>11 years old) and have yet to be substantiated; as such, these estimates may not reflect the state of the art.

The paucity of studies specifically exploring meditation utilization has also meant that there is little knowledge of the motivations, predictors and self-reported outcomes of meditation use. Addressing this knowledge gap may assist in furthering our understanding of the needs of health consumers, and the factors influencing health consumer behavior. Insights gained from such research will be valuable in informing practice, education and policy, as well as the future directions of meditation research. For these reasons, we set out to explore the lifetime and 12-month prevalence of meditation use, utilization patterns, and prevalence of specific meditation practices in the US general population by drawing upon 2012 National Health Interview Survey (NHIS) data. In addition, we aimed to identify the reasons for using meditation, the predictors of use, and the self-reported outcomes of meditation practice.

While predictors of meditation use have not been analyzed in prior studies, practices such as yoga, Tai chi and Qigong have been analyzed which share many similarities with meditation^11^,^12^. Based on those prior analyzes, we hypothesized meditation use to be associated with age, gender, ethnicity, area of residence, education, and health behavior.

## Methods

### Study design

Secondary analysis of 2012 US National Health Interview Survey (NHIS) data. The 2012 NHIS provides the most up-to-date version of the NHIS including information on mediation use.

### Data source

The analyses reported here were based on a nationally representative survey monitoring the health of the US population in 2012. More information on survey composition, sampling strategy, and administration of the NHIS can be found online (http://www.cdc.gov/nchs/nhis/about_nhis.htm). A total of 42,366 households were eligible to participate in the survey and 34,525 adults provided data (response rate: 79.7%)^13^. Population-based estimates were calculated using weights calibrated to the 2010 census-based population estimates for age, gender, and ethnicity of the US civilian non-institutionalized population.

Data from the NHIS Family Core, the NHIS Sample Adult Core, and the NHIS Adult Complementary and Alternative Medicine questionnaire were all used for our analyses.

The NHIS Family Core and the NHIS Sample Adult Core collected data on socio-demographic characteristics, including age, gender, ethnicity, region, marital status, education, and annual household income; as well as self-perceived general health status. The NHIS Adult Complementary and Alternative Medicine questionnaire collected data on the use of complementary and alternative medicine (CAM) therapies, including meditation. Lifetime meditation prevalence was determined by the following questions: *Have you EVER used any of the following for your own health or treatment? Mantra Meditation, including Transcendental Meditation^®^, Relaxation Response, and Clinically Standardized Meditation? Mindfulness meditation, including Vipassana, Zen Buddhist meditation, Mindfulness-based Stress Reduction, and Mindfulness-based Cognitive Therapy? Spiritual meditation including Centering Prayer and Contemplative Meditation?* Those who answered ‘Yes’ were presented with an additional question asking whether they had used these methods during the past 12-months.

Those who had practiced meditation in the past 12 months were asked to provide more details, such as whether they had attended a class of formal training, and the number and costs of attended classes. They were also questioned regarding their motivations for practicing meditation, the medical conditions for which they used meditation (from a total of 88 possible conditions), and the perceived benefit of meditation practice.

Yoga practice is assessed separately in the NHIS and not subsumed under meditation practice. The prevalence, patterns, and predictors of yoga use have thus not been included in this analysis but are available elsewhere^11^.

### Statistical analysis

Lifetime and 12-month prevalence of meditation use were analyzed descriptively, as were details on meditation, reasons for use and outcomes. Results were reported as means and standard deviations, weighted frequencies and distributions as eligible.

Sociodemographic characteristics were compared between those who had used meditation ever in their life/within the prior 12 months and those who had not using chi square tests. Independent predictors of meditation practice (i.e. ever used, used in previous 12 months) were identified using multiple logistic regression analysis. The following sociodemographic predictors were considered: age (categories: 18–29; 30–39; 40–49; 50–64, 65 years or older), gender (categories: female; male), ethnicity (categories: non-Hispanic White; Hispanic; African American; Asian; Other), region (categories: West; Northeast; Midwest; South), marital status (categories: not in relationship; in relationship), education (categories: less than college; some college or more), and annual household income (categories: less than US$20,000; US$20,000 to US$34,999; US$35,000-US$64,999; US$65,000 or more). Potential health-related predictors included general health status (categories: poor or fair; good, very good or excellent), body mass index (categories: <18.5; 18.5–25; 25–30; 30 kg/m^2^ or more), number of chronic conditions (categories: no chronic condition, one condition, more than one condition), and health behaviors such as smoking (categories: non-smoker, smoker), alcohol consumption (categories: alcohol abstainer; light drinker; regular or heavy drinker), and exercise behavior (categories: low level exerciser, moderate level exerciser, high level exerciser).

A backward stepwise procedure with a logistic regression statistic p-value of ≤0.05 was chosen, and adjusted odds ratios with 95% confidence intervals were calculated. Only those associated with meditation practice at a p-value of ≤0.10 (χ^2^-test) were included in the regression analyses. Statistical analyses were performed using the Statistical Package for Social Sciences software (IBM SPSS Statistics for Windows, release 22.0. Armonk, NY: IBM Corp).

## Results

### Prevalence of meditation use

Lifetime meditation prevalence for health reasons was 5.2%, representing 11.8 million US adults that had ever practiced meditation. Corresponding numbers for different types of meditation practices were 2.6% (5.8 million) for mantra, 2.5% (5.7 million) for mindfulness and 3.7% (8.3 million) for spiritual meditation practices. There were large overlaps in practice (i.e. 31.9% of users reported using two meditation practices and 17.9% reported using three meditation practices). Among those who had ever used meditation, 78.6% had practiced meditation within the past 12 months, representing 9.3 million or 4.1% of US adults. In the past 12 months, 1.6%, 1.9%, and 3.0% had practiced mantra, mindfulness, and spiritual meditation, respectively. Predictors for meditation practice in the past 12 months are presented in Table 1. Participants who practiced meditation were more likely to be female, aged between 40–64 years, non-Hispanic White, living in the Western US, have a higher education (college educated or above) and not be involved in a relationship. Participants who practiced meditation were more likely to suffer one or more chronic medical conditions, to be smokers, to not be alcohol abstinent and to be at least moderate level exercisers. Predictors of use were comparable across different types of meditation as were predictors for lifetime use and 12-month use (Supplementary Tables 1 and 2).

### Patterns of meditation practice

Among individuals who had used meditation in the past 12 months, 16.8% consulted a practitioner or participated in a class for meditation. The average number of visits in the past 12 months was 16.7 ± 17.9 (range: 1–52; median 8); at a total yearly cost of US$286.2 ± 559.4 (range: $0-6000; median $120). Only in 7.9% of cases costs were covered by health insurance. In addition, 19.9% of meditation users bought self-help books and other material to learn meditation, at a total cost of US$45.5 ± 55.5 (range: $0-200; median $20).

**Figure 1:**
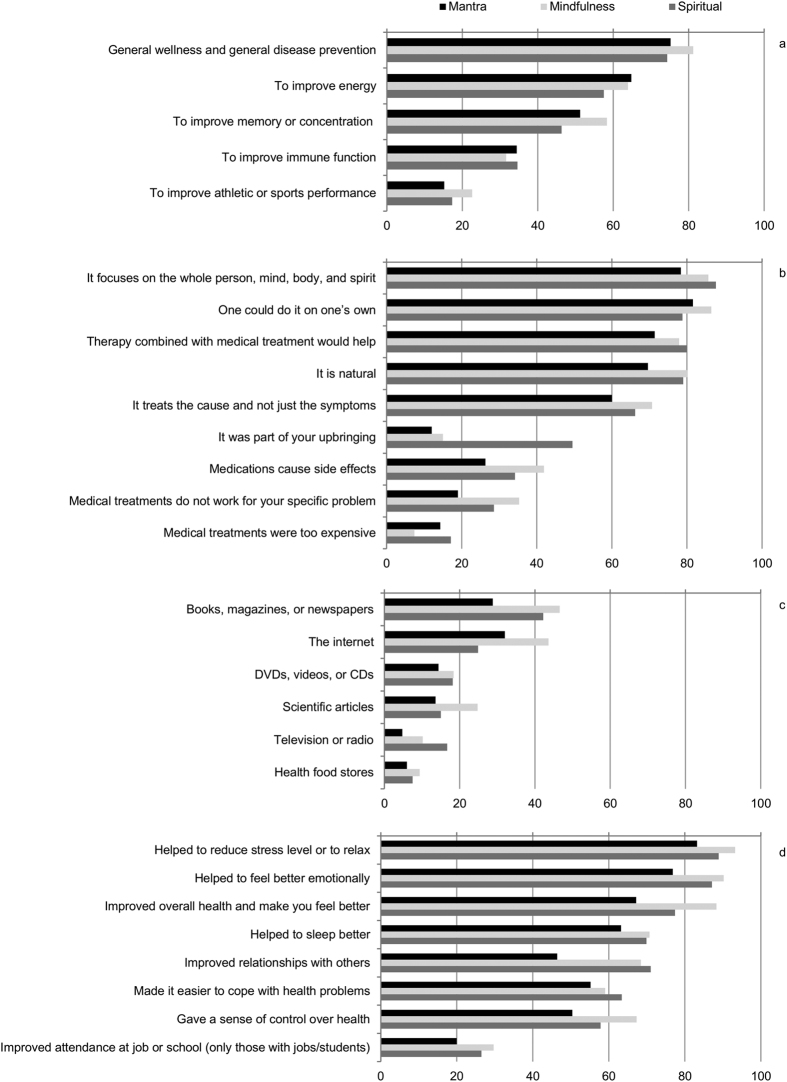
Reported reasons for using meditation (**a**,**b**); reported sources of information on meditation (**c**); and outcomes of using meditation (**d**) (% of respondents).

Many participants retrieved information about meditation from books, magazines or newspapers (41.7%), the Internet (30.6%), scientific articles (17.3%) and DVD’s and CDs (17.8%) among other sources (Table 2). Different information sources were utilized for different types of meditation practice, for example, seeking information from books, magazines and newspapers, the Internet and scientific articles was more strongly associated with the use of mindfulness meditation as compared to mantra or spiritual meditation (Fig. 1, Supplementary Table 3).

Most respondents reported practicing meditation for general wellness or disease prevention (76.2%), to improve their energy (60.0%), and/or to improve their memory or concentration (50.0%). Large percentages of respondents reported that meditation helped to reduce stress or to aid relaxation (89.4%), to feel better emotionally (86.9%), to improve overall health and make them feel better (79.0%) and/or to sleep better (69.3%) (Table 3). Adults who used mindfulness meditation self-reported positive outcomes more frequently when compared to adults who used mantra or spiritual meditation practices (Fig. 1, Supplementary Table 3).

Feeling anxious, nervous or worried (29.2%), experiencing frequent stress (21.6%), depression (17.8%) or back pain (12.0%) were the top specific health problems for which people practiced meditation, followed mainly by pain conditions and more unspecific health complaints such as insomnia or fatigue. Overall, 63.6% and 30.4% reported that meditation had helped a great deal or some deal to address these health problems, respectively (Table 3).

Meditation use was mainly recommended by friends (40.6%), and family members (28.9%), and less commonly by medical doctors (10.6%). Similarly, meditation use was disclosed to a personal health care provider by only 34.8% of users. Those who did not disclose their meditation use did so because their personal health provider did not explicitly ask about it (63.8%), the personal health provider was deemed by the respondent as not needing to know about their meditation use (58.3%) or not considered by the respondent to likely know as much as they did themselves about meditation (14.0%). Being worried about negative reactions or being discouraged to use meditation were infrequently reported as important reasons for non-disclosure of meditation use to a personal health provider (<3.1%).

## Discussion

Meditation is consistently one of the top five most commonly used CAM practices among US adults according to NHIS surveys conducted in 2002, 2007 and 2012^14^. As hypothesized, meditation use was associated with age, gender, ethnicity, area of residence, education, and health behavior.

Our analysis found the profile of a typical meditation user is similar to that of a general CAM user, namely being middle-aged, female, highly educated (college or higher education degree)^15^, non-Hispanic White and residing in a Western state of the US^16^.

The types of meditation used were reported in the NHIS for the first time in 2012 as mantra (2.6%), mindfulness (2.5%), or spiritual (3.7%). The nature of different types of meditation may contribute to the discrepancy in the frequency of use. For instance, spiritual meditation (for example centered prayer) may not require an instructor and can be done in a private setting while mindfulness meditation such as mindfulness-based stress reduction, or mantra meditation such as Transcendental Meditation^®^, often require guidance from a practitioner/instructor and accordingly, are often conducted in a teaching-learning setting^17^. Moreover, a larger number of US citizens might perceive prayer to fit with their (mainly Christian) worldview as compared to mindfulness or mantra meditation, which mainly derives from Buddhist and/or Hindu spiritual traditions. The majority of meditation users were found to practice more than one type of meditation indicating the three different types of meditation practice are not mutually exclusive. The effort of the NHIS to further explore the details of meditation using the three categories is important to understanding subtle differences in the contemporary use of meditation as applied meditations and mindfulness-based interventions show large differences in the way they are conceptualized and practiced^18^. The decision to consider such practices as unitary or as distinct phenomena will need to be considered carefully as it may influence the direction of future meditation research.

A wide range of instructional CDs, DVDs and books are available to teach and guide meditation practice. This is perhaps an explanation of why only a small proportion of meditation users in our sample reported attending meditation classes. Additionally, the Internet was accessed by one third of adults looking for information about mindfulness. With the increasing popularity of Internet-based applications (apps), accessed commonly through tablet devices and smartphones, it is conceivable that this use will grow. The potential impact of meditation apps remains largely unexplored^19^ and a recent review of 700 mindfulness-related apps identified from iTunes and Google Apps Marketplace, found that only 4% provided mindfulness training and education, while the rest were mostly guided meditation apps, timers, or reminders with poor Mobile App Rating Scale rating scores^20^. The lack of evidence for the effectiveness of mindfulness apps needs not only to be addressed in research but also in health education.

This study shows a higher proportion of adults who used meditation were individuals with anxiety, stress or depression. This is in line with research that demonstrates that patients living with chronic illness are attracted to complementary medicine self-care practices^21^. Emerging research demonstrates that meditation practices may be beneficial in the treatment of these psychological conditions^22^. A meta-analysis by Goyal and colleagues (2014) with a pooled sample size of 3515, reported mindfulness meditation had moderate effect sizes for anxiety, depression and pain; however, evidence was low for the effect of meditation on stress/distress and mental health quality of life measures^23^. A review by Marchand (2012) concluded two forms of meditation (mindfulness-based stress reduction and mindfulness-based cognitive therapy) had broad-spectrum effects by reducing depression, anxiety, and general psychological distress^22^.

In the clinical setting, depression and anxiety have been recognized as common consequences of chronic illness and research has shown that patients with chronic pain that suffer from anxiety and depression attain benefit from incorporating mindfulness meditation into their treatment plan^24^,^25^. A randomized controlled trial by la Cour and Petersen (2015) investigating the use of meditation for chronic pain demonstrated better quality of life in the mental health domain and better pain control for subjects practicing meditation^25^. Another study involving the use of meditation for patients receiving cancer treatment found that meditation resulted in lower scores for anxiety, depression and pain on post-intervention measures^26^. Outside the clinical setting, meditation has also been shown to benefit individuals with chronic psychological stress. For example, a study by Elder and colleagues (2014) reported individuals undertaking meditation improved on measures of perceived stress, depression and burnout symptoms. Thus the practice of meditation appears to be beneficial in environments where people have high levels of stress, such as the workplace^27^.

The reasons for meditation use and its perceived benefits found in this study support the broad appeal of meditation for its range of effects on physiology and mental function across groups, from those with depression and anxiety to those dealing with stress or chronic pain. The physiological benefits of meditation include lowering of blood pressure, decreasing cortisol levels, and improving active attention and emotion regulation in the cortex^28^. Furthermore, meditation affects the parasympathetic nervous system that induces relaxation leading to physiological and biochemical changes^28^,^29^. Studies have demonstrated that meditation increases cognitive function, decreases emotional reactivity, and enhances executive processes, which may help patients with depression and anxiety^29^. Further evidence of these physiological changes comes from neuroimaging studies that have shown increases in network efficiency and connectivity of the anterior cingulate cortex of the brain responsible for self-regulation^30^,^31^.

The growing evidence of the benefits of meditation, and its effects on physiological and psychological changes, has led to meditation becoming one of the more popular nonconventional therapies for a range of conditions, from depression/anxiety to chronic pain. Meditation practices, in both clinical and nonclinical settings, show promise in reducing anxiety, depression, and symptoms associated with psychological distress^32^. Unlike pharmacotherapy, which decreases neural network activity, meditation enhances brain network connectivity and regulation, which may influence disease progression and at the same time decrease stress, making meditation applicable for various chronic conditions and across populations (e.g. from clinical to nonclinical, different age groups, gender, etc.)^29^. Thus, more research is needed to determine the application and integration of sustainable meditation practice as an adjunct treatment for various chronic conditions. It may also be valuable to investigate whether self-taught meditation is as beneficial and sustainable as therapist-led practices.

The use of meditation for the relief of conditions such as depression, anxiety and chronic pain is a safe practice that is unlikely to negatively interact with other treatments. The side effects of meditation are extremely low or nonexistent; however, patients not discussing the use of meditation with their primary health provider may introduce a barrier to coordinated health care and interprofessional collaboration. The benefits of meditation may be overlooked by many conventional health providers due to its ‘hidden’ role in chronic health care in the community. The majority of participants that practiced meditation in this analysis did not disclose the use of this therapy to their health provider because they were not asked. Actively inquiring about the use of ‘other’ practices such as meditation could facilitate open, interprofessional collaboration and increase coordinated care for chronic health patients.

Interestingly, Asians living in the US were less likely to use meditation compared to non-Hispanic white US residents. This finding is relatively unexpected given that mantra meditation and mindfulness meditation derive from Hinduist and/or Buddhist tradition^33^ and thus originate from Asia. However, this might at least partly be explained by the fact that spiritual meditation was the category practiced most often, and this category included centering prayer which originally derives from Christian tradition^34^. Prior analyzes of the 2012 NHIS have shown that while Asians in the US were also less likely to practice yoga as compared to non-Hispanic Whites^11^, they were more likely to practice Tai Chi or Qigong^12^.

Other minority populations were also less likely to use meditation, as were seniors. Cardiovascular risk is higher in minorities^35^ and the risk increases with age^36^. Given that meditation has been shown to decrease cardiovascular risk factors such as hypertension, type 2 diabetes mellitus, and dyslipidemia^37^, examining reasons for non-use of meditation among minority populations and elderly US residents seems worthwhile given that these populations might likely benefit from meditation.

### Limitations

First, the data are part of a cross-sectional survey; therefore, the results can only indicate correlations, not causal effects. The interpretations that can be drawn from the findings are strengthened, however, by the regression analysis, which controls for confounding variables. Second, data were obtained by self-report, thus limiting the findings of this analysis. To elaborate, participants were asked to recall the details of their use of meditation practices over the previous 12 months; as such, the data may be affected by recall bias. Finally, the available data were limited. Besides ethnicity, culture can be expected to strongly influence the prevalence and patterns of meditation use and while this survey only included US residents, assessing self-perceived cultural links of the participants might have improved analysis of predictors and patterns. However, cultural links were not assessed in the NHIS. Notwithstanding, the opportunity to analyze data from a large, nationally representative sample largely counters these concerns.

## Conclusions

The use of meditation by patients with chronic health conditions is perhaps unsurprising as mind-body health practices have long been associated with more holistic approaches to chronic health care. This research does, however, shed more light on the characteristics of the meditation user. It also points to a largely self-managed, self-taught and ‘hidden’ health intervention, with few users disclosing meditation use to a health care professional. These findings have potential implications for clinical practice, meditation training and patient/clinician education.

## Additional Information

**How to cite this article**: Cramer, H. *et al*. Prevalence, patterns, and predictors of meditation use among US adults: A nationally representative survey. *Sci. Rep*. **6**, 36760; doi: 10.1038/srep36760 (2016).

**Publisher’s note**: Springer Nature remains neutral with regard to jurisdictional claims in published maps and institutional affiliations.

## Supplementary Material

Supplementary Information

## Figures and Tables

**Table 1:** Predictors associated independently with meditation use in the last 12 months among adults in the US (n = 9,296,917; 4.1%).

	OR (95% CI)	p
Age		
18–29	Reference	
30–39	1.25 (1.05; 1.48)	0.010
40–49	1.06 (0.89; 1.27)	0.484
50–64	1.34 (1.14; 1.57)	<0.001
65+	0.66 (0.53; 0.82)	<0.001
Gender		
Male	Reference	
Female	1.52 (1.37; 1.69)	<0.001
Ethnicity		
Non-Hispanic White	Reference	
Hispanic	0.67 (0.56; 0.81)	<0.001
Black	0.63 (0.52; 0.76)	<0.001
Asian	0.58 (0.45; 0.74)	<0.001
Other	1.16 (0.71; 1.90)	0.551
Region		
West	Reference	
Northeast	0.56 (0.48; 0.65)	<0.001
Midwest	0.61 (0.54; 0.70)	<0.001
South	0.48 (0.42; 0.55)	<0.001
Education		
Less than high school	Reference	
High school	2.11 (1.63; 2.73)	<0.001
Some college or more	4.53 (3.50; 5.87)	<0.001
Marital status		
not in relationship	Reference	
in relationship	0.74 (0.65; 0.82)	<0.001
BMI		
18.5 to 25	Reference	
up to 18.5	0.86 (0.57; 1.30)	0.466
25–30	0.87 (0.77; 0.98)	0.025
30 and more	0.76 (0.66; 0.86)	<0.001
Multiple chronic conditions		
no chronic condition	Reference	
1 chronic condition	1.40 (1.24; 1.59)	<0.001
2 chronic conditions	1.75 (1.48; 2.08)	<0.001
3 or more chronic conditions	1.51 (1.20; 1.88)	<0.001
Health behaviour		
Smoking		
Non smoking	Reference	
Smoking	1.24 (1.09; 1.42)	0.002
Alcohol consumption		
Abstainers	Reference	
Light	1.46 (1.28; 1.66)	<0.001
Moderate to heavy	1.60 (1.38; 1.87)	<0.001
Exercise		
Low level exerciser	Reference	
Moderate level exerciser	1.41 (1.14; 1.60)	<0.001
High level exerciser	1.38 (1.11; 1.70)	0.003

Variables included in final regression model: age, gender, ethnicity, region, education, marital status, BMI, number of chronic conditions, smoking, alcohol consumption, exercise behavior.

**Table 2:** Reasons for using meditation, health care provider interaction, and information sources.

	% of meditation users
Meditation was used because	
Medical treatments were too expensive	14.0
Therapy combined with medical treatment would help	78.6
Medical treatments do not work for your specific health problem	29.7
Medications cause side effects (only those who received medication)	35.9
One could do it on one’s own	81.1
It is natural	78.1
It focuses on the whole person, mind, body, and spirit	86.1
It treats the cause and not just the symptoms	66.6
It was part of your upbringing	36.1
Meditation was recommended by	
A medical doctor	10.6
A family member	28.9
A friend	40.6
A co-worker	9.5
Meditation practice disclosed to personal health care provider	34.8
Not disclosed because	
Not used at the time	12.6
Being worried they would discourage it	2.6
Being concerned about a negative reaction	3.1
Didn’t think they needed to know	58.3
They didn’t ask	63.8
Don’t think they know as much about it as you do	14.0
They didn’t give enough time to tell them	8.6
Information source	
The internet	30.6
Books, magazines, or newspapers	41.7
DVDs, videos, or CDs	17.8
Television or radio	13.6
Scientific articles	17.3
Health food stores	7.8

Note: Categories with less than 2% responders are not shown due to reduced certainty in the weighing process.

**Table 3:** Associations between meditation practice and health.

	% of meditation users
Reasons to use meditation	
For general wellness or general disease prevention	76.2
To improve energy	60.0
To improve immune function	33.8
To improve athletic or sports performance	18.4
To improve memory or concentration	50.0
Meditation helped with the most important reason	
A great deal	65.0
Some	29.2
Only a little	5.5
Meditation motivated to	
Eat healthier	34.9
Exercise more regularly	34.1
Cut back or stop drinking alcohol (only those who drink alcohol)	13.2
Cut back or stop smoking cigarettes (only those who smoke)	7.1
Eat more organic food	21.8
Meditation led to	
Gave a sense of control over health	59.3
Helped to reduce stress level or to relax	89.4
Helped to sleep better	69.3
Helped to feel better emotionally	86.6
Made it easier to cope with health problems	61.3
Improved overall health and make you feel better	79.0
Improved relationships with others	67.4
Improved attendance at job or school (only students/employed)	26.5
Used meditation for a specific health problem (top health problem)	
Feeling anxious, nervous or worried	29.2
Frequent stress	21.6
Depression	17.8
Back pain	12.0
Joint pain	9.6
Insomnia, trouble sleeping	9.4
Severe headache or migraine	9.4
Fatigue, lack of energy	7.9
Chronic pain	7.6
Neck pain	7.0
Muscle or bone pain	6.1
Mental health disorders, others	5.3
Cancer	5.3
Hypertension	4.7
Meditation helped for specific health problem	
A great deal	63.6
Some	30.4
Only a little	4.2

Note: Categories with less than 2% responders are not shown due to reduced certainty in the weighing process.
