# Supplementary material for: Prevalence, patterns, and predictors of meditation use among US adults: A nationally representative survey
Source: Sci Rep. 2016 Nov 10;6:36760. doi: 10.1038/srep36760 (PMC5103185; doi:10.1038/srep36760)
Supplement: Supplementary Information [file srep36760-s1.pdf]

Title

Prevalence, patterns, and predictors of meditation use among US adults: A nationally representative survey

Supplement

Authors

Holger Cramer, PhD[1,2*], Helen Hall PhD[2,3], Matthew Leach PhD[2,4], Jane Frawley PhD[2], Yan Zhang PhD[2,5], Brenda Leung PhD[2,6], Jon Adams PhD[2], Romy Lauche PhD[2]

Affiliation

[1] Department of Internal and Integrative Medicine, Kliniken Essen-Mitte, Faculty of Medicine, University of Duisburg-Essen, Essen, Germany.

[2] Australian Research Centre in Complementary and Integrative Medicine (ARCCIM), Faculty of Health, University of Technology Sydney, Sydney, New South Wales, Australia.

[3] School of Nursing and Midwifery, Monash University, Frankston, VIC, Australia.

[4]School of Nursing & Midwifery, University of South Australia, Adelaide, South Australia.

[5] Department of Family and Community Medicine, Texas Tech University Health Sciences Center, Lubbock, Texas, USA.

[6] University of Lethbridge, Lethbridge, Alberta, Canada.

Supplementary Table 1: Predictors associated independently with having ever used meditation among adults in the US.

Weighted using sample size adjusted weights (in order not to increase the power by pretending to have more cases)	Ever practice of any meditation<br><br>OR (95% CI)<br><br>n=11,822,115<br>5.2%	Ever practice of Mantra meditation<br><br>OR (95% CI)<br><br>n=5,818,300<br>2.6%	Ever practice of mindfulness meditation<br>OR (95% CI)<br><br>n=5,720,809<br>2.5%	Ever practice of spiritual meditation<br><br>OR (95% CI)<br><br>n=8,303,367<br>3.7%
Age				
18-29	Reference	Reference	Reference	Reference
30-39	1.42 (1.20; 1.68)	1.74 (1.36; 2.22)	1.32 (1.05; 1.66)	1.49 (1.22; 1.82)
40-49	1.22 (1.03; 1.45)	1.58 (1.23; 2.03)	1.18 (0.93; 1.50)	1.31 (1.07; 1.61)
50-64	1.51 (1.29; 1.78)	2.04 (1.62; 2.58)	1.49 (1.19; 1.85)	1.59 (1.31, 1.93)
65+	0.70 (0.57; 0.87)	0.93 (0.69; 1.26)	0.53 (0.39; 0.72)	0.77 (0.60. 0.90)
Gender				
Male	Reference	Reference	Reference	Reference
Female	1.49 (1.34; 1.66)	1.21 (1.04; 1.40)	1.41 (1.21; 1.63)	1.50 (1.33; 1.70)
Ethnicity				
Non-Hispanic White	Reference	Reference	Reference	Reference
Hispanic	0.66 (0.55; 0.79)	0.59 (0.45; 0.78)	0.51 (0.39; 0.67)	0.69 (0.56; 0.86)
Black	0.62 (0.51; 0.75)	0.68 (0.52; 0.89)	0.45 (0.32; 0.62)	0.68 (0.55; 0.85)
Asian	0.59 (0.46; 0.76)	0.57 (0.40; 0.81)	0.53 (0.38; 0.75)	0.62 (0.46; 0.84)
Other	1.14 (0.70; 1.87)	1.32 (0.70; 2.50)	0.83 (0.38; 1.79)	1.12 (0.63; 1.99)
Region				
West	Reference	Reference	Reference	Reference
Northeast	0.60 (0.52; 0.70)	0.60 (0.49; 0.73)	0.43 (0.35; 0.53)	0.61 (0.51; 0.73)

Midwest	0.64 (0.56; 0.73)	0.57 (0.47; 0.69)	0.51 (0.43; 0.62)	0.69 (0.59; 0.81)
South	0.49 (0.43; 0.56)	0.44 (0.36; 0.53)	0.41 (0.34; 0.49)	0.56 (0.48; 0.65)
Education				
Less than college	Reference	Reference	Reference	Reference
Some college or more	3.34 (2.94; 3.90)	3.64 (2.96; 4.47)	3.29 (2.68; 4.06)	3.07 (2.62; 3.61)
Marital status				
not in relationship	Reference	Reference	Reference	Reference
in relationship	0.76 (0.69; 0.85)	0.76 (0.65; 0.88)	0.76 (0.66; 0.92)	0.70 (0.62; 0.80)
Health status				
excellent/very good	Reference	Reference	Reference	Reference
good	0.98 (0.86; 1..11)	0.94 (0.79; 1.12)	1.09 (0.91; 1.30)	0.86 (0.74; 0.99)
fair/poor	1.39 (1.17; 1.66)	1.42 (1.11; 1.80)	1.56 (1.21; 2.01)	1.18 (0.96; 1.44)
BMI				
18.5 to 25	Reference	Reference	Reference	-
up to 18.5	0.89 (0.59; 1.34)	1.41 (0.87; 2.29)	1.00 (0.59; 1.70)	
25-30	0.88 (0.78; 0.99)	0.76 (0.65; 0.92)	0.78 (0.66; 0.92)	
30 and more	0.76 (0.67; 0.87)	0.72 (0.60; 0.87)	0.52 (0.43; 0.64)	
Multiple chronic conditions				
no chronic condition	Reference	Reference	Reference	Reference
1 chronic condition	1.39 (1.22; 2.00)	1.45 (1.22; 1.72)	1.32 (1.11; 1.57)	1.41 (1.22; 1.63)
2 chronic conditions	1.69 (1.42; 1.78)	1.66 (1.31; 2.09)	1.40 (1.09; 1.80)	1.72 (1.41; 2.09)
3 or more chronic conditions	1.43 (1.14. 1.40)	1.31 (0.96; 1.80)	1.17 (0.83; 1.65)	1.48 (1.14; 1.90)
Health behaviour				
Smoking				

Non smoking	Reference	Reference	-	Reference
Smoking	1.23 (1.07; 1.40)	1.28 (1.06; 1.53)		1.20 (1.03; 1.40)
Alcohol consumption				
Abstainers	Reference	Reference	Reference	Reference
Light	1.50 (1.32; 1.70)	1.67 (1.39; 2.01)	1.83 (1.50; 2.20)	1.34 (1.15; 1.55)
Moderate to heavy	1.65 (1.42; 1.92)	1.78 (1.44; 2.21)	2.13 (1.71; 2.65)	1.31 (1.10; 1.57)
Exercise				
Low level exerciser	Reference	Reference	Reference	Reference
Moderate level exerciser	1.40 (1.24; 1.59)	1.38 (1.16; 1.65)	1.44 (1.21; 1.71)	1.12 (1.11; 1.52)
High level exerciser	1.37 (1.11; 1.70)	1.58 (1.20; 2.07)	1.43 (1.07; 1.90)	1.21 (0.94; 1.57)

Supplementary table 2: Predictors associated independently with mantra meditation, mindfulness meditation, and spiritual meditation use in the last 12 months among adults in the US who had ever used meditation.

Weighted using sample size adjusted weights (in order not to increase the power by pretending to have more cases)	12 months Mantra meditation practice<br>OR (95% CI)<br>n=3,611,989<br>1.6%	12 months mindfulness practice meditation<br>OR (95% CI)<br>n=4,314,339<br>1.9%	12 months spiritual meditation practice<br>OR (95% CI)<br>n=6,907,775<br>3.0%
Age			
18-29	Reference	Reference	Reference
30-39	1.39 (1.04; 1.85)	1.20 (0.92; 1.55)	1.45 (1.16; 1.80)
40-49	1.10 (0.81; 1.49)	1.12 (0.86; 1.46)	1.41 (1.13; 1.76)
50-64	1.37 (1.04; 1.81)	1.34 (1.06; 1.70)	1.59 (1.29; 1.96)
65+	0.69 (0.48; 0.99)	0.51 (0.37; 0.72)	0.72 (0.55; 0.94)
Gender			
Male	Reference	Reference	Reference
Female	1.44 (1.20; 1.73)	1.33 (1.12; 1.57)	1.63 (1.42; 1.86)
Ethnicity			
Non-Hispanic White	Reference	Reference	Reference
Hispanic	0.59 (0.42; 0.83)	0.42 (0.30; 0.59)	0.61 (0.47; 0.77)
Black	0.70 (0.51; 0.98)	0.49 (0.34; 0.70)	0.73 (0.58; 0.92)
Asian	0.70 (0.46; 1.08)	0.47 (0.32; 0.71)	0.56 (0.40; 0.79)
Other	0.69 (0.24; 1.99)	0.76 (0.30; 1.89)	1.23 (0.67; 2.25)
Region			
West	Reference	Reference	Reference

Northeast	0.57 (0.44; 0.74)	0.43 (0.34; 0.55)	0.68 (0.56; 0.83)
Midwest	0.62 (0.49; 0.78)	0.52 (0.42; 0.64)	0.74 (0.62; 0.88)
South	0.48 (0.38; 0.61)	0.39 (0.32; 0.49)	0.62 (0.52; 0.73)
Education			
Less than college	Reference	Reference	Reference
Some college or more	3.41 (2.64; 4.39)	3.29 (2.59; 4.18)	3.23 (2.70; 3.86)
Marital status			
not in relationship	Reference	Reference	Reference
in relationship	0.71 (0.59; 0.85)	0.75 (0.63; 0.89)	0.68 (0.59; 0.77)
Health status	-	Reference	-
excellent/very good			
good		1.17 (0.96; 1.43)	
fair/poor		1.58 (1.19, 2.10)	
BMI			
18.5 to 25	-	Reference	-
up to 18.5		1.21 (0.70; 2.09)	
25-30		0.82 (0.68; 0.99)	
30 and more		0.53 (0.43; 0.67)	
Multiple chronic conditions			
no chronic condition	Reference	-	Reference
1 chronic condition	1.62 (1.31; 2.00)		1.42 (1.21; 1.66)
2 chronic conditions	1.58 (1.18; 2.12)		1.68 (1.37; 2.73)
3 or more chronic conditions	1.44 (0.98. 2.10)		1.59 (1.23; 2.06)
Health behaviour			

Smoking			
Non smoking	Reference	-	-
Smoking	1.39 (1.12; 1.73)		
Alcohol consumption			
Abstainers	Reference	Reference	Reference
Light	2.25 (1.76; 2.89)	1.80 (1.44; 2.24)	1.26 (1.07; 1.47)
Moderate to heavy	2.39 (1.80; 3.17)	1.85 (1.44; 2.38)	1.32 (1.10; 1.60)
Exercise			
Low level exerciser	-	Reference	Reference
Moderate level exerciser		1.48 (1.22; 1.80)	1.31 (1.11; 1.55)
High level exerciser		1.53 (1.11; 2.10)	1.25 (0.94; 1.65)

Supplementary table 3: Associations between mantra meditation, mindfulness meditation, and spiritual meditation practice and health, reasons for using the respective type of meditation, health care provider interaction, and information sources.

	Mantra meditation	Mindfulness meditation	Spiritual meditation
Reasons to use meditation			
For general wellness or general disease prevention	75.2	81.2	74.3
To improve energy	64.8	63.9	57.5
To improve immune function	34.4	31.6	34.6
To improve athletic or sports performance	15.2	22.6	17.3
To improve memory or concentration	51.2	58.3	46.3
Meditation helped with the most important reason			
A great deal	51.3	60.0	69.7
Some	40.2	33.1	25.4
Only a little	8.5	6.5	4.6
Meditation motivated to …			
Eat healthier	22.4	37.6	36.3
Exercise more regularly	28.0	37.6	33.8
Cut back or stop drinking alcohol (only those who drink alcohol)	9.6	14.7	13.2
Cut back or stop smoking cigarettes (only those who smoke)	5.6	6.8	7.5
Eat more organic food	14.4	25.9	21.5
Meditation led to …			
Gave a sense of control over health	50.4	67.3	57.8
Helped to reduce stress level or to relax	83.2	93.2	88.9
Helped to sleep better	63.2	70.7	69.9
Helped to feel better emotionally	76.8	90.2	87.1
Made it easier to cope with health problems	55.2	59.0	63.4
Improved overall health and make you feel better	67.2	88.3	77.4

Improved relationships with others	46.4	68.4	71.0
Improved attendance at job or school (only students/employed)	20.0	29.7	26.5
Used meditation for a specific health problem (top health problem)			
Feeling anxious, nervous or worried	29.0	30.4	28.8
Frequent stress	22.6	20.7	21.9
Depression	22.6	21.7	15.5
Back pain	-	9.8	14.6
Joint pain	9.7	5.4	11.4
Insomnia, trouble sleeping	6.5	5.4	11.4
Severe headache or migraine	6.5	6.5	11.0
Fatigue, lack of energy	6.5	3.3	10.0
Chronic pain	16.1	8.7	5.9
Neck pain	-	3.3	9.6
Muscle or bone pain	6.5	2.2	7.8
Mental health disorders, others	3.2	5.4	5.5
Cancer	6.5	2.2	6.4
Hypertension	9.7	2.2	5.0
Meditation helped for specific health problem …			
A great deal	51.6	55.4	68.9
Some	38.7	37.0	26.4
Only a little	6.5	6.5	2.8
Not at all	3.2	-	0.9
Received the following for specific health problem			
Prescription medication	51.6	53.3	47.6
OTC medication	22.6	28.3	25.0
Surgery	6.5	8.7	13.2
Physical therapy	9.7	10.9	16.5
Mental health counselling	35.5	44.6	31.1
Meditation was used because …			

Medical treatments were too expensive	14.3	7.4	17.1
Therapy combined with medical treatment would help	71.4	77.9	80.0
Medical treatments do not work for your specific health problem	19.0	35.3	28.6
Medications cause side effects (only those who received medication)	26.3	41.9	34.2
One could do it on one’s own	81.6	86.5	78.8
It is natural	69.6	80.1	79.0
It focuses on the whole person, mind, body, and spirit	78.4	85.7	87.7
It treats the cause and not just the symptoms	60.0	70.7	66.2
It was part of your upbringing	12.0	15.0	49.5
Meditation was recommended by …			
A medical doctor	14.4	15.0	8.1
A family member	19.2	21.8	33.8
A friend	41.6	38.7	41.1
A co-worker	9.6	8.6	9.8
Meditation practice disclosed to personal health care provider	37.0	28.2	36.8
Not disclosed because …			
Not used at the time	14.3	22.8	8.8
Being worried they would discourage it	-	3.3	2.9
Being concerned about a negative reaction	1.8	2.2	3.7
Didn’t think they needed to know	64.3	62.0	55.9
They didn't ask?	64.3	63.0	64.0
Don't think they know as much about it as you do	14.3	17.4	12.9
They didn't give enough time to tell them	5.4	12.0	8.1
Information source			
The internet	32.0	43.6	24.9
Books, magazines, or newspapers	28.8	46.6	42.2
DVDs, videos, or CDs	14.4	18.4	18.2

Television or radio	4.8	10.2	16.7
Scientific articles	13.6	24.8	15.0
Health food stores	6.0	9.4	7.5
Note: Categories with less than 2% responders are not shown due to reduced certainty in the weighing process.			
